# The electronic cigarette: a knight in shining armour or a Trojan horse?†

**DOI:** 10.1192/pb.bp.114.048439

**Published:** 2014-10

**Authors:** Neil W. Schluger

**Affiliations:** 1 World Lung Foundation, Columbia University College of Physicians and Surgeons and Mailman School of Public Health, New York

## Abstract

Electronic cigarettes have caused a sharp debate in the public health community, with some promoting them as a means of harm reduction for tobacco users and some taking a strong stand against them because of fear of renormalising smoking behaviour and interrupting tobacco control progress. People with mental health problems smoke at high rates and e-cigarettes seem a potentially attractive method of cessation in this population, and their use should be studied carefully.

Nothing has quite roiled the waters of the anti-smoking community in the past several years as has the appearance of electronic cigarettes (e-cigarettes). At almost precisely the same moment, when a consensus has finally emerged that smoked tobacco represents an almost uniquely harmful threat to public health - it is after all, the world’s leading preventable cause of death - and when tougher and tougher anti-smoking measures have penetrated the smokiest of smoke-filled rooms (no more smoking in public houses, even), e-cigarettes have managed to split the public health community in two: those who view them as a potentially valuable harm reduction tool to reduce the morbidity and mortality associated with smoking tobacco on the one side, and those who view them as a Trojan horse designed to renormalise smoking behaviour, addict a new generation of young people to nicotine, and to slow the anti-tobacco momentum that has gathered slowly but powerfully across the globe. At this point, the evidence is mixed and not definitive. The stakes are high in this argument, and for people with mental illness, they may be higher still, as the article by Ratschen^1^ in this issue points out.

## The two sides of the argument

The argument from the harm reduction community basically states that we must accept the fact that among a certain percentage of the population, smoking is inevitable, and the only thing that we can do as health advocates for this segment of the population is to make smoking safer. E-cigarettes represent a much safer alternative than tobacco, this argument continues, because they remove what seem to be the most dangerous components in traditional cigarettes - the hundreds of carcinogens and other toxins produced by tobacco that are presumably responsible for cancers of the aerodigestive tract, emphysema, heart disease and the long list of other maladies that cigarettes cause. E-cigarettes satisfy the craving for nicotine without the other toxins that tobacco products contain: hence, harm reduction.

On the other side of the argument are those who view e-cigarettes as a real threat to the progress that has been made in the past few years in decreasing tobacco use. In cities such as New York, aggressive measures to raise cigarette prices to as much as US$12.50 (nearly £7.50) per pack, to severely limit public smoking (including a ban on tobacco use in parks and on beaches), and to raise the legal age for purchase of cigarettes to 21 years have driven smoking rates down to just 14% of the population. This is a remarkable achievement and has doubtless saved lives, and many who have worked hard to secure these successes oppose widespread e-cigarette use. In fact, the local New York City government recently voted to include e-cigarettes in the general ban on smoking in public places. Advocates for this position make a two-pronged argument. First, they feel that widespread use of e-cigarettes, many of which look almost identical to traditional tobacco cigarettes, will renormalise smoking behaviour, particularly among young people, by creating a glamorous image associated with use of the electronic devices. This will threaten tobacco control activities that have successfully convinced people that smoking is a socially unacceptable and undesirable behaviour. The second component to this argument holds that e-cigarettes in fact might remove incentives to quit tobacco, because they will allow smokers to bridge their use of tobacco at home and in private with electronically delivered nicotine while in public or at work. The proliferation of e-cigarettes could provide further excuses for governments not to implement measures such as those used in New York City and elsewhere and to delay or avoid full implementation of the Framework Convention on Tobacco Control. The Framework Convention on Tobacco Control has been endorsed by most governments in the world, although its full and specific provisions have been implemented by relatively few countries.

At this point, where does the balance of the evidence stand, with those opposed to e-cigarettes or with those who view them as potentially helpful in reducing disease from tobacco use?

## Electronic cigarettes as a harm reduction device

Harm reduction is a strategy that rests on the belief or understanding that certain unhealthy behaviours will never be eliminated and the goal should move to reducing bad effects that accompany these behaviours. This strategy has been advocated most notably and effectively in relation to intravenous drug use and the risk of HIV infection, and indeed this approach has achieved some success.^2^ The use of needle exchange programmes to reduce the transmission of HIV infection is supported by good evidence. In addition, fears that provision of free syringes would lead to increased drug use have not been substantiated by experience. By extension, use of e-cigarettes could reduce harmful effects of tobacco without encouraging use generally. Up to now, harm reduction from smoking cigarettes has been limited primarily to the use of products such as smokeless tobacco, and there is no significant evidence that these products have provided any public health benefit. Could e-cigarettes be more effective?

Notably, e-cigarette manufacturers have chosen not to develop and market their products primarily as smoking cessation or harm reduction devices, although they could have done so. In 2009, the United States Food and Drug Administration (FDA) announced that it would attempt to regulate e-cigarettes as medical devices, which would have forced manufacturers to demonstrate that e-cigarettes were safe and effective in harm reduction. The FDA was then sued successfully by a group of manufacturers who said that e-cigarettes were specifically not drugs, drug delivery systems or drug device combinations, and that they should be regulated instead as tobacco products. Thus, given the chance to develop their products as health related or medically useful devices, manufacturers chose not to. E-cigarette advertising campaigns developed subsequently have positioned these devices as variously glamorous, sophisticated, sexually attractive, or macho, but never as therapeutic or designed to reduce harm from tobacco. Thus, the major thrust of the industry’s efforts has been the development of a new market in nicotine addiction rather than creation of a therapy to lower harm from tobacco use. To be sure, local advocates for unrestricted e-cigarette use always testify that they have used them to reduce their tobacco dependency, but one suspects that this is just a clever strategy by which companies seek to eat their cake and have it too.

If there were ever an industry that does not deserve the benefit of the doubt when it comes to protecting or promoting the public’s health, it is the tobacco industry, and one notes with alarm that Big Tobacco has moved quickly into the e-cigarette market. Reynolds, Lorillard, British American Tobacco and Altria (i.e. Phillip Morris) have all taken major stakes in e-cigarette manufacturing. However, just because e-cigarette manufacturers are primarily interested in selling nicotine addiction does not mean that careful use of their product might not have some benefit for smokers, and that is the question before us.

### Long-term safety of e-cigarettes

Before e-cigarettes can be endorsed or even studied on a large scale as harm reduction devices, basic questions about their safety should be answered. Although registration in the USA as drugs or drug delivery devices would have subjected them to the ‘safe and effective’ standard, the US FDA does have some authority to regulate them as tobacco products. Indeed, the agency recently announced its intention to do so. Evidence about the safety of e-cigarettes to date is scant and mixed. Nicotine is a highly addictive substance, so once people begin to use e-cigarettes they may find it difficult to stop. A recent study suggested that e-cigarette use may decline over time, although patterns of e-cigarette use seem to be affected by prior or current tobacco consumption.^3^

Although it is likely that nicotine is not a carcinogen in and of itself,^4^ there is abundant evidence that nicotine has substantial effects on many organ systems, and the long-term effects of nicotine must be studied. At present, no long-term studies have been done, and such research is sorely needed. As the current draft guidance from the UK’s National Institute For Health And Care Excellence (NICE) states, ‘there is no evidence on the long-term safety of e-cigarettes, whether used alone or with concurrent cigarette smoking’.^5^ Reflecting this, the European Parliament has gone quite a bit further than the US government, and sales and advertising are quite restricted in the European Union. Legislation passed in the European Parliament in 2014 bans e-cigarette advertising, regulates the nicotine content in the devices and requires graphic warning labels on packaging. These restrictions are scheduled to go into effect in 2016.

Although most studies that have examined the effect of smoking on the lungs have used ‘whole’ tobacco smoke, there are some reports that examine the effects of nicotine alone. A recent paper by Maouche and colleagues in France demonstrated that chronic nicotine exposure in mice was associated with defects in mucus transport that mimicked changes found in patients with chronic obstructive pulmonary disease and cystic fibrosis.^6^ Moreover, the effects of nicotine on the developing lung can be profound. A recent study demonstrated that germline epigenetic changes induced by perinatal nicotine exposure were associated with the development of asthma-like responses in the lungs of several generations of mice,^7^ and prior work has shown that nicotine has significant effects on fetal lung development.^7-12^ The relevance of these studies in humans is unclear, but it seems cavalier at this point to say that there is no risk to the lungs from chronic nicotine exposure.

## Electronic cigarettes as an aid to smoking cessation

And yet. Although smoking rates are falling in some places, and restrictions on smoking are more widespread throughout Europe and elsewhere, there are still hundreds of millions of tobacco smokers in the world, and quitting is very difficult, although the benefits of doing so are great.^13,14^ A typical smoker will make many, many attempts to quit in his or her lifetime, and most of these will fail. As the article by Ratschen^1^ makes clear, the damage done by tobacco smoking, particularly to patients with mental illness, is considerable, and there are few effective methods of smoking cessation or harm reduction in this patient population. Although I strongly support strict limitations on the general marketing, sale and use of e-cigarettes in the population at large, given the imperative to identify strategies to reduce tobacco use and the harm done by tobacco smoke to patients with mental illness, this seems an ideal opportunity for well-designed clinical trials that could look at the effectiveness of e-cigarettes as smoking cessation devices and their short- and long-term safety. Studies regarding the utility of e-cigarettes as smoking cessation aids to date are mixed. Many surveys indicate that smokers give high marks to e-cigarettes in their attempts to quit tobacco,^15,16^ but the largest and most rigorously designed studies indicate that e-cigarettes are associated with low sustained quit rates that are not much higher than other forms of nicotine replacement therapy,^17^ It is high time to start settling this issue with good science. Efficacy and short-term safety in people with mental illness could be easily and well studied with randomised trials, and this should be an imperative. Long-term safety will have to be assessed through carefully done observational studies, and waiting until these longer term studies are done does not seem fair to those in urgent need of smoking cessation. We should proceed, but with great caution.
